# Factors associated with unrealized fertility among women approaching the end of reproductive age in sub-Saharan Africa

**DOI:** 10.1371/journal.pone.0331265

**Published:** 2025-09-02

**Authors:** Tsion Mulat Tebeje, Mesfin Abebe, Achamyeleh Birhanu Teshale, Mekonnen Birhanie Aregu

**Affiliations:** 1 School of Public Health, College of Health Science and Medicine, Dilla University, Dilla, Ethiopia; 2 Department of Midwifery, College of Health Science and Medicine, Dilla University, Dilla, Ethiopia; 3 Department of Epidemiology and Biostatistics, Institute of Public Health, College of Medicine and Health Sciences, University of Gondar, Gondar, Ethiopia; 4 Department of Environmental Health, School of Public Health, College of Health Science and Medicine, Dilla University, Dilla, Ethiopia; Babol University of Medical Sciences, IRAN, ISLAMIC REPUBLIC OF

## Abstract

**Background:**

Understanding women’s fertility preferences is essential for addressing reproductive behaviors and family planning needs. In sub-Saharan Africa, fertility rates remain high, yet many women experience unrealized fertility, which is having fewer children than desired. However, the factors influencing unrealized fertility remain underexplored. This study assessed the determinants of unrealized fertility among women approaching the end of their reproductive years in sub-Saharan Africa.

**Methods:**

A secondary data analysis was conducted using phase eight Demographic and Health Surveys data from 19 sub-Saharan African countries. The weighted sample included 46,408 women aged 40–49 years. A multilevel Poisson regression model with robust variance was used to identify factors associated with unrealized fertility. Adjusted prevalence ratios with 95% confidence intervals (CI) were reported, and variables with a p value <0.05 were considered statistically significant.

**Results:**

The pooled prevalence of unrealized fertility among women aged 40–49 years was 61.43% (95% CI: 57.63%, 65.24%). Rwanda (37.40; 95% CI: 27.92%, 46.88%) and Sierra Leone (69.34; 95% CI: 60.30%, 78.38%) had the lowest and highest prevalence, respectively. Older maternal age at first birth, being employed, having no children or only children of one sex, and experiencing child death were associated with higher prevalence of unrealized fertility. Conversely, higher maternal education, the use of contraceptives, having both male and female children, and residing in rural areas were associated with lower prevalence of unrealized fertility.

**Conclusions:**

A large proportion of women nearing the end of their reproductive careers in sub-Saharan Africa have experienced unrealized fertility. Therefore, addressing cultural norms surrounding sex preference and number of children, alongside empowering women through improved access to education, healthcare, and comprehensive sexual and reproductive health services, is critical.

## Introduction

Fertility is a key factor in population dynamics that affects the size and growth of populations [1]. Globally, fertility rates have been declining, with the current rate standing at 2.25 live births per woman, which is one child less than in 1990. However, women in sub-Saharan Africa (SSA) countries have an average of four or more births, and the region’s population growth remains high. The population of SSA is projected to rise from 1.1 billion in 2022 to over 2 billion by 2050 and 3.44 billion by 2100. Although the average number of births per woman in SSA is expected to decline from 4.6 in 2021 to 3.0 in 2050 [2,3], fertility remains above replacement levels. Additionally, women in SSA experience higher levels of unrealized fertility and fertility desires [4–6]. These SSA women have more difficulty translating their fertility preferences into birth outcomes compared to women in other low- and middle-income countries (LMICs) [7].

Fertility desires and intentions impact a woman’s fertility decisions, choices, behavior, and achievements at different stages of her life [8]. Fertility is considered achieved when the number of children a woman desires matches the number she have. Conversely, unrealized fertility occurs when a woman has fewer children than she desires. In essence, unrealized fertility refers to the situation in which women in the later stages of their reproductive years are unable to meet their ideal fertility goals [4,5].

Unrealized fertility is more prevalent in LMICs, such as SSA, North Africa, Latin America, and South and Southeast Asia [4]. Within these, SSA experiences notably higher rates, with 40% of women facing unrealized fertility compared to 26% in non-SSA countries. Furthermore, unrealized fertility varies across African regions, with western and central Africa exhibiting the highest rates, followed by eastern and southern Africa [6,9–11].

Achieving the desired level of fertility requires individuals to have control over their reproductive decisions. That is, people have the right to make their own decisions about their fertility preferences without interference. However, not all women are equally vulnerable to failure to reach fertility goals, suggesting that certain factors contribute to unrealized fertility. A handful of studies have assessed these factors that potentially contribute to the occurrence of unrealized fertility in LMICs, including SSA [4,11]. These studies indicated that higher levels of education, higher wealth quintiles, and contraceptive use as are associated with unrealized fertility. In contrast, the impact of factors such as experiencing child death and the working status of women on unrealized fertility remains under explored.

While most research in SSA has focused on high fertility rates and the unmet need for contraception, it is important to examine women who wish to have more children but are unable to reach their desired family size. Exploring the factors that contribute to unrealized fertility can help understand reproductive behaviors and family planning needs. This leads to the development of effective family planning initiatives that support women in achieving their desired family size, while also respecting individual reproductive rights and enhancing overall societal well-being. We investigated unrealized fertility among women aged 40–49 in SSA, who are approaching the end of their reproductive years, making them an appropriate group for comparing the number of their living children and fertility preferences [10]. This study assessed the factors influencing unrealized fertility in 19 SSA countries, aiming to provide new insights into why intentions do not always translate into reality.

## Methods

### Data source and sampling procedure

**Fig 1:**
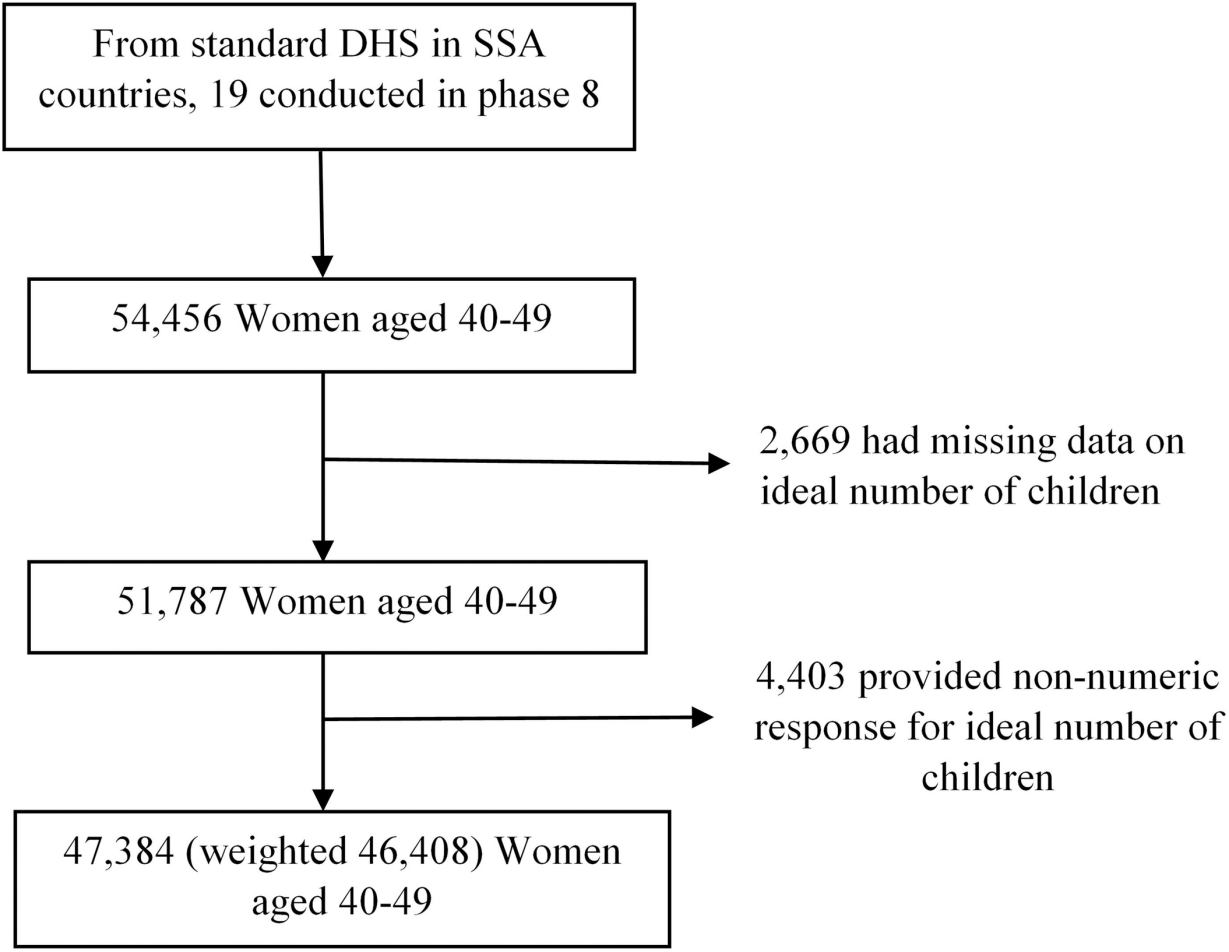
Flowchart of the study.

This study is based primarily on 19 SSA countries that have conducted their most recent demographic and health surveys (DHS), not including the mini-DHS, in phase 8 (2018–2023) to have the same available variables and up-to-date data. Within each country, the cross-sectional survey used a two-stage cluster sampling technique. The first step involved the random selection of enumeration areas (EAs), whereas the second stage involved the selection of households. The Individual Women’s Record dataset (IR file) was used. The DHS survey data from the 19 SSA nations were combined, and a weighted sample of 46,408 women between the ages of 40 and 49 was included [12]. The flow chart illustrating how the study samples were selected is depicted in Fig 1.

### Variables of the study

**Dependent variable**: Unrealized fertility was the outcome variable. According to Casterline and Han [13], unrealized fertility can be measured via two indicators: (i) the ideal number of children and (ii) prospective fertility desire for another child. The 2^nd^ indicator has major limitations since it is limited to women who are in union, who are fecund, and who decide to want another child. Therefore, in this study, the 1^st^ indicator was used to measure unrealized fertility. The ideal number of children was self-reported and explored through two questions [13]:

1) “If you could choose exactly the number of children to have in your whole life, how many would that be?” (for respondents with no children); and
2) “If you could go back to the time you did not have any children and could choose exactly the number of children to have in your whole life, how many would that be?” (for respondents who had children).

Then, women whose ideal number of children exceeds their current number of living children were categorized as having unrealized fertility; otherwise, they were categorized as having realized fertility.

**Independent variables**: The independent variables were selected from the literature [4,10,11,14,15] on the basis of their presence in the DHS datasets. These variables include residence (categorized as urban and rural), sex of the household head (male and female), marital status (never married, currently married/partnered, and widowed/divorced/separated), education level (no formal education, primary, secondary, and higher), wealth index (poorest, poorer, middle, richer, or richest), occupation (employed and not employed), age at first birth (<20, 20–24, 25–29, and 30+), number of living children (0–2, 3, 4, 5, and 6+), sex composition (no child, all sons, all daughters, equal, more sons, more daughters, and no child), experiencing child death (yes and no), contraceptive utilization (none, traditional, or modern), and exposure to family planning messages (yes and no).

### Data management and analysis

With STATA V.17, data cleaning, recoding, and analysis were carried out. After appending the data from the 19 SSA countries, the data were weighted. The pooled prevalence of unrealized fertility among women aged 40–49 years was analyzed via the STATA command “metan” and was displayed in a forest plot. Owing to the hierarchical nature of the DHS data, a multilevel analysis was conducted. Additionally, considering that the data were collected cross-sectional and given the high prevalence of unrealized fertility, which was 61.43%, reporting odds ratio could exaggerate the relationship between the dependent and independent variables. For the aforementioned reasons, a multilevel Poisson regression with robust variance was used, and the prevalence ratio was reported [16,17]. The variance inflation factor (VIF) was used to determine if there was multicollinearity among the independent variables, and multicollinearity was less likely since the VIF was less than five for all the variables. To explore the factors associated with unrealized fertility, four multilevel Poisson regression models with robust variance were built. Model 1, fitted using the outcome variable only, was employed to ascertain the degree of variability of unrealized fertility between clusters via the intracluster correlation coefficient (ICC). Individual-level factors were used to fit the second model (Model 2), community-level variables were considered in the third model (Model 3), and both individual- and community-level variables were used concurrently to fit the fourth model (Model 4). The best fitted model was selected on the basis of deviance, and the model with the lowest deviance value was considered in the final model. Variables with p values < 0.2 in the bi-variable analysis were eligible for the multivariable analysis. Finally, adjusted prevalence ratios (aPR) with 95% confidence intervals (CI) were reported, and statistical significance was determined at a p value < 0.05.

### Ethical approval and consent to participate

The data were obtained from the Demographic and Health Surveys (DHS) Program and can be freely accessed from the website (www.dhsprogram.com). Since this study involved a secondary data analysis of publicly available data from the MEASURE DHS program, ethical approval and participant consent were not necessary for this particular study. We obtained permission from the DHS Program to access and use the data for our study. The dataset was downloaded from https://dhsprogram.com/data/available-datasets.cfm. The procedures approved by the IRB for DHS public-use datasets do not allow for the identification of respondents, households, or sample communities.

## Results

### Background characteristics of the participants

Among the total respondents, 36,410 (78.5%) were currently married and 20,079 (43.3%) had never attended formal education. Furthermore, approximately half of the participants (n = 22584, 48.7%) had exposure to family planning messages, and the majority (n = 26,883, 57.9%) were rural dwellers. Among the participants, (n = 21,103, 47%) gave birth for the first time before the age of 20, and three-quarters of the women were not using any form of contraceptive. With respect to family size, (n = 22,704, 48.9%) of the respondents’ ideal number of children was 6 + , (n = 15,515, 33.4%) had 6 or more living children, (n = 16,294, 35.1%) experienced child death, (n = 8,250, 17.8%) had an equal number of sons and daughters, (n = 14,366, 31%) had more sons, and (n = 13,737, 29.6%) had more daughters (Table 1).

**Table 1:** Background characteristics and distribution of unrealized fertility of women at the end of their reproductive period in sub-Saharan Africa.

Variables		Weighted Frequency (%)	Unrealized fertility	
			Yes (%)	No (%)
**Age in years**	40–44	25,741 (55.5)	15,808 (61.4)	9,933 (38.6)
	45–49	20,668 (44.5)	12,562 (60.8)	8,106 (39.2)
**Working status**	Not working	10,711 (23.6)	6,532 (61.0)	4,179 (39.0)
	Working	34,692 (76.4)	21,140 (60.9)	13,552 (39.1)
**Education level**	No formal education	20,079 (43.3)	13,286 (66.2)	6,793 (33.8)
	Primary	14,731 (31.7)	7,995 (54.3)	6,736 (45.7)
	Secondary	9,128 (19.7)	5,467 (59.9)	3,662 (40.1)
	Higher	2,470 (5.3)	1,621 (65.6)	849 (34.4)
**Marital status**	Single	1,764 (3.8)	1,309 (74.2)	456 (25.8)
	Married/living with partner	36,410 (78.5)	21,889 (60.1)	14,522 (39.9)
	Widowed/divorced/separated	8,235 (17.7)	5,173 (62.8)	3,062 (37.2)
**Contraceptive utilization**	None	35,247 (76.0)	23,342 (66.2)	11,905 (33.8)
	Traditional	1,694 (3.6)	809 (47.8)	885 (52.2)
	Modern	9,468 (20.4)	4,219 (44.6)	5,250 (55.4)
**Wealth index**	Poorest	8,618 (18.6)	5,490 (63.7)	3,127 (36.3)
	Poorer	8955 (19.3)	5,515 (61.6)	3,439 (38.4)
	Middle	9172 (19.8)	5,400 (58.9)	3,772 (41.1)
	Richer	9336 (20.1)	5,537 (59.3)	3,799 (40.7)
	Richest	10,328 (22.2)	6,426 (62.2)	3,901 (37.8)
**Exposure to family planning message**	No	23,825 (51.3)	15,007 (63.0)	8,817 (37.0)
	Yes	22,584 (48.7)	13,362 (59.2)	9,222 (40.8)
**Ideal number of children**	0–3	7326 (15.8)	2,056 (28.1)	5,270 (71.9)
	4–5	16,379 (35.3)	8,465 (51.7)	7,914 (48.3)
	≥6	22,704 (48.9)	17,848 (78.6)	4,855 (21.4)
**Age at first birth**	< 20	21,103 (47.0)	12,072 (57.2)	9,031 (42.8)
	20–24	14,839 (33.1)	8,483 (57.2)	6,356 (42.8)
	25–29	5,973 (13.3)	3,980 (66.6)	1,994 (33.4)
	≥30	2,979 (6.6)	2,415 (81.1)	564 (18.9)
**Number of living children**	0–2	9,170 (19.8)	8,329 (90.8)	841 (9.2)
	3	6,558 (14.1)	5,092 (77.6)	1,466 (22.3)
	4	7,737 (16.7)	4,616 (59.7)	3,121 (40.3)
	5	7,428 (16.0)	3,956 (53.2)	3,473 (46.7)
	≥6	15,515 (33.4)	6,377 (41.1)	9,138 (58.9)
**Sex composition**	No child	1,830 (3.9)	1731 (94.6)	99 (5.4)
	All sons	4,161 (9.0)	3473 (83.5)	688 (16.5)
	All daughters	4,064 (8.8)	3353 (82.5)	710 (17.5)
	Equal	8,250 (17.8)	4941 (59.9)	3,309 (40.1)
	Son > daughter	14,366 (31.0)	7622 (53.1)	6,744 (46.9)
	Daughter > son	13,737 (29.6)	7248 (52.8)	6,489 (47.2)
	No child	1,830 (3.9)	1731 (94.6)	99 (5.4)
**Experienced child death**	No	30,115 (64.9)	17335 (57.6)	12,780 (42.4)
	Yes	16,294 (35.1)	11,034 (67.7)	5,259 (32.3)
**Sex of household head**	Male	32,109 (69.2)	19,455 (60.6)	12,653 (39.4)
	Female	14,300 (30.8)	8,914 (62.3)	5,386 (37.7)
**Residence**	Urban	19,526 (42.1)	12,244 (62.7)	7282 (37.3)
	Rural	26,883 (57.9)	16,125 (60.0)	10,757 (40.0)

### Family size and fertility preferences

The mean ideal number of children was 5.8, which ranges from 4.2 in Rwanda to 6.7 in Nigeria and Mali. The mean age at first birth was 20.8 years, which was highest in Rwanda (22.5 year) and lowest in Liberia and Zambia (19.5 year). The mean numbers of children ever born, living children, and dead children in SSA were 5.2 (ranging from 4.0 in Gabon to 6.5 in Mali), 4.5 (ranging from 3.8 in Gabon to 5.4 in Mali), and 0.65 (ranging from 0.25 in Gabon to 1.13 in Mali), respectively (Table 2).

**Table 2:** Mean of fertility preference and family size of women at the end of their reproductive period in sub-Saharan Africa by country.

Country	Mean				
	Ideal number of children	Age at first birth (in year)	Children ever born	Living children	Dead children
**SSA**	5.8	20.8	5.2	4.5	0.65
Burkina Faso	6.5	20.7	5.6	4.9	0.64
Cote d’Ivoire	6.0	20.6	4.9	4.3	0.57
Cameroon	6.4	20.2	5.3	4.6	0.74
Gabon	5.2	20.4	4.0	3.8	0.25
Ghana	5.2	21.7	4.5	4.1	0.36
Gambia	6.4	19.9	5.7	5.1	0.59
Guinea	5.9	21.4	4.9	4.2	0.68
Kenya	4.3	20.8	4.3	4.0	0.34
Liberia	5.7	19.5	5.2	4.3	0.93
Madagascar	5.2	20.8	4.8	4.3	0.50
Mali	6.7	20.5	6.5	5.4	1.13
Mauritania	6.5	22.8	5.2	4.8	0.37
Mozambique	5.6	20.5	4.9	4.4	0.53
Nigeria	6.7	20.4	6.1	5.0	1.06
Rwanda	4.2	22.5	4.9	4.3	0.62
Sierra Leone	5.9	19.9	5.2	4.2	1.01
Senegal	5.5	22.4	4.8	4.4	0.35
Tanzania	6.1	20.2	5.1	4.6	0.47
Zambia	5.9	19.5	5.8	5.1	0.71

### Pooled prevalence of unrealized fertility

**Fig 2:**
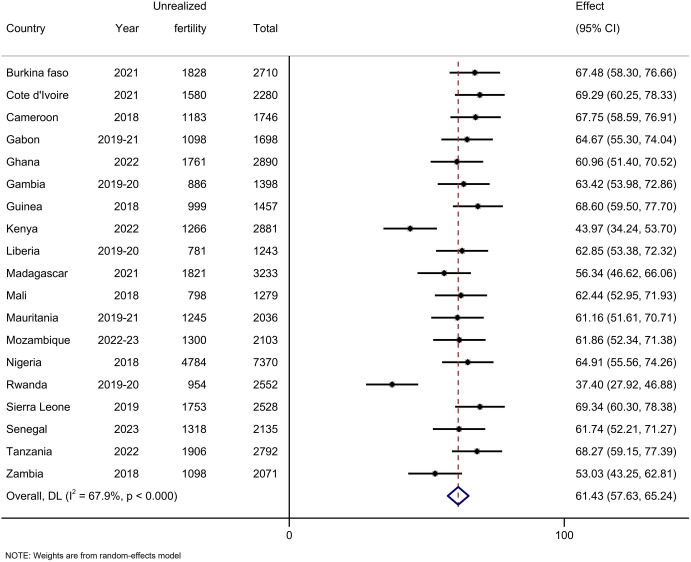
Forest plot of the pooled prevalence of unrealized fertility among women at the end of their reproductive period in sub-Saharan Africa.

The pooled prevalence of unrealized fertility was 61.43% (95% CI: 57.63, 65.24), in which Rwanda (37.40; 95% CI: 27.92, 46.88) and Sierra Leone (69.34; 95% CI: 60.30, 78.38) had the lowest and highest prevalence, respectively (Fig 2). The prevalence of unrealized fertility was higher among women whose ideal number of children was more than six (78.6%), whose age at first birth was 30 year or above (81.1%), whose all children were of the same sex (83.5% for all sons and 82.5% for all daughters), and those who had experienced child death (67.7%) than their counterparts (Table 1).

### Factors associated with unrealized fertility

The ICC value in the null model was 13.26% (95% CI; 12.15%, 14.47%), indicating that 13.26% of the overall variability in unrealized fertility resulted from between-cluster variability, whereas the remaining 86.74% of the overall variability was attributable to individual variation. Therefore, to obtain robust estimates in the presence of this clustering effect, a multilevel model was considered. In the bi-variable analysis, the sex of the household head, education level, wealth index, age at first birth, marital status, contraceptive utilization, exposure to family planning messages, working status, sex composition, experienced child death, and residence status were selected with p values <0.2. In the multivariable multilevel robust Poisson regression analysis, educational status, age at first birth, contraceptive utilization, working status, sex composition, experiencing child death, and residence were significantly associated with unrealized fertility.

The prevalence of experiencing unrealized fertility among women who reached primary, secondary, and higher education decreased by 15% [aPR = 0.85, 95% CI: 0.83, 0.87], 11% [aPR = 0.89, 95% CI: 0.87, 0.91], and 11% [aPR = 0.89, 95% CI: 0.85, 0.92], respectively, compared with women who had no formal education. Women aged 20–24 years, 25–29 years, and 30 years and above at their first birth had 1.05 times [aPR = 1.05, 95% CI: 1.04, 1.08], 1.20 times [aPR = 1.20, 95% CI: 1.17, 1.22], and 1.31 times [aPR = 1.31, 95% CI: 1.28, 1.34] higher prevalence of unrealized fertility, respectively, than women aged under 20 years. Women who were working were 1.03 times more likely [aPR = 1.03, 95% CI: 1.01, 1.05] to experience unrealized fertility compared to women who were not working. Compared with not utilizing any contraceptives, the use of traditional and modern contraceptives decreased the prevalence of experiencing unrealized fertility by 23% [aPR = 0.77, 95% CI: 0.73, 0.81] and 26% [aPR = 0.74, 95% CI: 0.72, 0.76], respectively. Compared with women who had an equal number of sons and daughters, women who had no child, women who had only sons, and women who had only daughters had a 1.31 [aPR = 1.31, 95% CI: 1.27, 1.35], 1.34 [aPR = 1.34, 95% CI: 1.30, 1.37], and 1.33 [aPR = 1.33, 95% CI: 1.30, 1.37] times higher prevalence of experiencing unrealized fertility, respectively. In contrast, women who have more sons than daughters and more daughters than sons had 10% [aPR = 0.90, 95% CI: 0.88, 0.92] and 11% [aPR = 0.89, 95% CI: 0.87, 0.91], respectively, decreased prevalence of unrealized fertility. The prevalence of having unrealized fertility among women who experienced child death increased by 25% [aPR = 1.25, 95% CI: 1.23, 1.27]. Compared with women from urban areas, the prevalence of unrealized fertility among women from rural areas decreased by 3% [aPR = 0.97, 95% CI: 0.95, 0.99] (Table 3).

**Table 3:** Assessment of factors associated with unrealized fertility of women at the end of their reproductive period in sub-Saharan Africa (N = 46,408).

Variable	Null model	Model 2	Model 3	Model 4
		aPR (95%CI)	aPR (95%CI)	aPR (95%CI)
**Sex of household head**				
Male		1		1
Female		0.99 (0.97, 1.01)		0.99 (0.97, 1.00)
**Education level**				
No education		1		1
Primary		0.85 (0.84, 0.87)		0.85 (0.83, 0.87)***
Secondary		0.89 (0.87, 0.91)		0.89 (0.87, 0.91)***
Higher		0.89 (0.85, 0.92)		0.89 (0.85, 0.92)***
**Wealth index**				
Poorest		1		1
Poorer		1.00 (0.98, 1.02)		0.99 (0.97, 1.02)
Middle		0.98 (0.96, 1.01)		0.98 (0.96, 1.00)
Richer		1.00 (0.98, 1.03)		0.98 (0.96, 1.02)
Richest		1.02 (0.99, 1.05)		1.00 (0.97, 1.04)
**Age at first birth**				
< 20		1		1
20-24		1.06 (1.04, 1.07)		1.05 (1.04, 1.08)**
25-29		1.19 (1.17, 1.22)		1.20 (1.17, 1.22)***
30+		1.31 (1.28, 1.34)		1.31 (1.28, 1.34)***
**Marital status**				
Single		1		1
Married/living with partner		0.97 (0.93, 1.01)		0.97 (0.93, 1.01)
Widowed/divorced/separated		0.98 (0.94, 1.02)		0.98 (0.94, 1.02)
**Contraceptive utilization**				
None		1		1
Traditional		0.76 (0.73, 0.81)		0.77 (0.73, 0.81)***
Modern		0.74 (0.72, 0.76)		0.74 (0.72, 0.76)***
**Exposure to family planning message**				
No		1		1
Yes		0.99 (0.98, 1.01)		0.99 (0.98, 1.01)
**Working status**				
Not working		1		1
Working		1.03 (1.01, 1.04)		1.03 (1.01, 1.05)***
**Sex composition**				
Equal		1		1
No child		1.31 (1.27, 1.35)		1.31 (1.27, 1.35)***
All sons		1.34 (1.30, 1.37)		1.34 (1.30, 1.37)***
All daughters		1.33 (1.30, 1.37)		1.33 (1.30, 1.37)***
Son > daughter		0.90 (0.88, 0.92)		0.90 (0.88, 0.92)***
Daughter > son		0.89 (0.87, 0.91)		0.89 (0.87, 0.91)***
**Experienced child death**				
No		1		1
Yes		1.25 (1.23, 1.27)		1.25 (1.23, 1.27)***
**Residence**				
Urban			1	1
Rural			0.97 (0.96, 0.99)	0.97 (0.95, 0.99)*
**ICC (%)**	13.26 (12.15, 14.47)			
**Log-Likelihood**	−43174.55	−39600.21	−43172.45	−39598.79
**Deviance**	86349.1	79200.42	86344.9	79197.58

aPR = adjusted prevalence ratio

CI = confidence interval

* p value <0.05

** p value <0.01

*** p value <0.001

## Discussion

It has been agreed internationally that parents have the right to decide freely and responsibly on the number, timing, and spacing of their children [18,19]. In this context, our study explores the determinants of unrealized fertility in SSA. The pooled prevalence was high, and we found sociodemographic, reproductive, and contextual factors influencing unrealized fertility, including education level, age at first birth, contraceptive utilization, working status, sex composition of children, experiencing child death, and place of residence.

The pooled prevalence of unrealized fertility among women aged 40–49 years in SSA was 61.43 (95% CI: 57.63, 65.24). This was in line with a report from a previous study in LMICs [20], where unrealized fertility was 62% in western and central Africa. A related study conducted in Nigeria found that the prevalence of unrealized fertility was 13.5% when measured by the indicator “desire for another child.” However, when the indicator “ideal number of children” was used as the measurement, the prevalence rose significantly to 59.2%, which aligns with our findings [21]. A study revealed that 36% of women in Ghana experienced underachieved fertility [15], which is lower than our findings. This disparity arises from variations in how the outcome variable is measured. Our outcome variable was measured by the difference between the ideal number of children and the number of living children, as stated by Casterline and Han [13]. However, the Ghanaian study calculated the difference between the ideal number of children and the total number of children ever born to measure underachieved/unrealized fertility [4].

According to our results, women who reported having no children, only son/s, or only daughter/s were more likely to report a higher prevalence of experiencing unrealized fertility. In contrast, the possibility of having unrealized fertility was less prevalent among women who have both sons and daughters of unequal numbers than among those with equal numbers of sons and daughters. Parental preference for the sex composition of children is one of the factors that influences fertility [21–24], particularly in LMICs, where a strong preference for sons or daughters affects the likelihood of having more children. Gender preferences vary widely across different countries and regions, with balance preference being the most common type globally. However, the daughter preference was more prevalent in Latin America, the Caribbean, and some Southeast Asian countries, whereas the son preference was dominant in southern Asia, western Asia, and northern Africa [25]. In SSA, there is little evidence of sex preference, primarily a mix of son and daughter preferences, with son preference being more common in Western Africa and daughter preference in other regions [11,25]. A qualitative study from Brazil revealed that couples’ desire for children varies based on the number of children they have, where most desire more than what they have and having children of each sex [26]. Our finding shows that while women may prefer to have both sons and daughters rather than children of only one sex, the actual number of sons and daughters they have is not as influential as long as they have children of both sexes and achieve their desired total number of children.

As the age of a woman at her first birth is delayed to older ages, it is more likely to have a higher prevalence of unrealized fertility. This finding is in line with other previous studies [4,21]. Women are born with a finite number of eggs, which decrease in both quality and quantity as they age. Fertility declines in women in their early 30s, notably after age 35, with a 44% chance of conceiving within a year by age 40. Therefore, an older age at childbearing is characterized by decreased chances of conception, increased risks of miscarriage and pregnancy complications, and longer times to achieve pregnancy [27,28]. Consistent with studies performed in Nigeria [14] and Ghana [15], a high prevalence of unrealized fertility was more likely to be experienced among women who experienced child death. A possible explanation could be that in SSA, where there is high child mortality and a desire for larger family sizes (based on the results presented in Table 2), the discrepancy between desire and reality leads to underachieved fertility.

Women who used any method of contraceptive were less likely to attain unrealized fertility than their counterparts. This is supported by studies from LMICs [11,20]. Family planning helps achieve the desired family size because women use contraception to control their family size after they reach their ideal number of children [29]. In agreement with other studies [11,14,20], the more educated the mother is, the less likely she is to have unrealized fertility than mothers with no formal education. This is because educated women tend to have a more limited number of ideal family sizes and living children. In contrast, women with no formal education may have larger ideal family sizes, which leads to a greater likelihood of unrealized fertility if they cannot achieve those numbers. Additionally, those with lower levels of education are also more likely to experience unwanted fertility and pregnancies [30]. Women who are employed tend to have lower total fertility rates and a lower unmet need for family planning, and are more likely to use modern contraceptives compared to those who are not employed [31]. In support of this, our results revealed a statistically significant association between women’s working status and unrealized fertility. Women who reported that they were working or employed were more likely to have a higher prevalence of experiencing unrealized fertility.

Women residing in rural areas were found to have a lower prevalence of unrealized fertility than urban residents. This finding contradicts findings from other studies [11,14]. The difference may be due to variations in the age of study populations and the survey years. Those studies brought lack of resources in rural areas to support their findings. However, women in rural areas often have higher total fertility rates resulting from lower levels of education, economic conditions, and cultural norms that favor larger families compared to their urban counterparts. Additionally, lower employment status and poor knowledge of contraceptives contribute to higher fertility in rural areas [32–34]. Therefore, the lower likelihood of unrealized fertility among rural residents might be attributable to high fertility.

### Strengths and limitations of this study

This study was based on a pooled nationally representative DHS of 19 SSA nations that increase generalizability and can be used as the foundation for appropriate evidence-based decisions. As a caveat, because DHS data are cross-sectional, we were unable to infer cause-effect linkages. The ideal number of children and other fertility-related data are based on women’s self-reports, which makes this study prone to social desirability and recall bias. Additionally, the heterogeneity of the pooled estimate of unrealized fertility was not managed by further analysis.

## Conclusion

This study found that unrealized fertility is notably prevalent among women approaching the end of their reproductive period in sub-Saharan Africa. Older maternal age at first birth, being employed, having no children or only children of one sex, and experiencing child death contributed to higher prevalence of unrealized fertility . In contrast, higher maternal education, the use of contraceptives, having both male and female children, and residing in rural areas contributed to lower prevalence. Therefore, interventions targeting empowering women through education and finance contribute to deciding on the number of their children freely. Additionally, efforts to expand women’s access to education, family planning, and healthcare, alongside initiatives that challenge societal norms on sex preference and family size, are essential.

## Abbreviations

aPR: adjusted prevalence ratio
CI: confidence interval
DHS: demographic and health survey
ICC: intracluster coefficient
LMICs: low- and middle-income countries
SSA: sub-Saharan Africa
VIF: variance inflation factor
